# High-Intensity Focused Electrical Stimulation and Sync Radiofrequency+ Technology for Submental Volume Reduction: An MRI Study

**DOI:** 10.1093/asj/sjaf048

**Published:** 2025-04-03

**Authors:** Barry DiBernardo, Carolyn Jacob, Lesley Clark-Loeser

## Abstract

**Background:**

Excessive weight gain, aging-related skin laxity, and weakened digastric muscle contribute to the formation of submental fullness.

**Objectives:**

The aim of this study was to investigate the efficacy and safety of combined high-intensity focused electrical stimulation (HIFES) and novel Synchronized Radiofrequency+ (Sync RF+) energies for submental volume reduction, and in addition to examine the effect of HIFES and Sync RF treatment on fat volume in the cheek area.

**Methods:**

Thirty-three subjects (n = 33) received 4 treatments once weekly, on the submental and cheek area. Two- and three-dimensional photographs were taken at baseline, after the fourth treatment, and at both follow-ups at 1 and 3 months posttreatment. MRI scanning was performed at baseline, and at both follow-ups.

**Results:**

The overall submental volume decreased by 25.12% at 1 month and by 36.20% at 3 months. The submental fat decreased by 20.54% at 1 month and by 30.37% at 3 months. The mean [standard deviation] volume reduction evaluated from 3D photography was 3.48 [3.60] mL immediately after the fourth treatment, 5.39 [5.93] mL at 1 month, and 10.25 [5.40] mL at 3 months. The mean Clinician-Reported Submental Fat Rating Scale grade improved by 0.56 [0.42] points after the fourth treatment, by 0.85 [0.53] points at 1 month, and by 1.03 [0.50] points at 3 months. Overall, 84.8% of subjects found the treatment comfortable and 93.9% were satisfied with the treatment results.

**Conclusions:**

The study findings suggest that this novel approach offers a notable option for submental volume reduction, indicating that HIFES and Sync RF+ technology is capable of aesthetic enhancement as well as skin and muscle improvement.

**Level of Evidence: 4 (Therapeutic):**

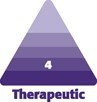

Submental fullness, colloquially termed as a double chin, diminishes facial definition, resulting in an undesired appearance and a decrease in confidence, leading to psychological discomfort in some social situations, such as being photographed.^1^ Various interacting factors contribute to the formation of submental fullness, ranging from genetic predispositions and aging, to lifestyle choices.^2^ Excessive weight gain, aging-related skin laxity, and weakened digastric muscle are among the primary instigators^2-4^. The decline in elasticity and atrophy in the dermis with age ultimately leads to skin laxity, which, coupled with the gravitational pull, results in prominence in the submental area.^3^ Another factor involved in submental fullness is the anterior belly of the digastric muscle (ABDM). McCleary et al noted that this muscle decreases in volume with advancing age, and suggested that loss of muscle tone and subsequent protrusion cause increased submental convexity.^4,5^ Additionally, genetic predispositions influence the distribution of fat deposits, making some individuals more susceptible to developing a double chin.^2^ With aging and genetic predisposition being part of the causation, the reduction of a double chin through lifestyle modifications can be quite challenging and sometimes futile.^6^

Therefore, multiple techniques have emerged for submental fullness reduction, both surgical and nonsurgical. Although invasive liposuction or surgical neck lifts show great efficacy, their downtime and possible adverse events^7,8^ can dissuade patients from opting for these treatments. Even nonsurgical interventions can carry the risk of irreversible adverse effects. For instance, deoxycholic acid injections, a method frequently utilized to reduce submental fat, have been associated with several reported complications, including nerve injury, alopecia, skin necrosis, edema, and bruising.^9^ However, nonsurgical approaches generally offer shorter downtimes and lower risks of adverse events but may also be insufficiently effective as they target either the lax skin or fat tissue while neglecting the muscle tissue.^10^

A technology incorporating high-intensity focused electrical stimulation (HIFES) energy combined with skin rejuvenation synchronized radiofrequency (Sync RF) energy has emerged as an established method for facial rejuvenation, particularly in the forehead and midfacial region^11-13^. This technology has been expanded to treat the submental area using Synchronized Radiofrequency+ (Sync RF+), a novel variant, in addition to the Sync RF used in facial treatments. Sync RF+ delivers higher power specifically designed to target excessive fat tissue in the submental region, while still preserving the skin rejuvenation benefits associated with Sync RF.

The application of radiofrequency for skin rejuvenation has been widely investigated, and increased collagen and elastin production has been demonstrated.^14,15^ Additionally, existing aged fibers undergo a structural reorganization to a formation resembling that of younger fibers.^16^ The benefits for fat tissue after radiofrequency exposure include its reduction through the hyperthermic induction of apoptosis.^17,18^ HIFES technology targets the weakened ABDM by stimulating the mylohyoid nerve. This electricity-based energy induces supramaximal muscle contractions, similar to intense exercise, which has been shown to activate heat-shock proteins and quiescent satellite cells, and the muscle stem cells—the elements responsible for muscle regeneration^19-21^. It is hypothesized that improved ABDM quality leads to reversion of the protrusion. While HIFES energy stimulates the digastric muscle, Sync RF+ targets the skin and subcutaneous fat tissue concurrently. Together, HIFES and Sync RF+ technology aims to reduce the submental volume by simultaneously decreasing digastric muscle protrusion and excessive fat tissue, while improving skin laxity.

This study investigates the influence of a novel method combining HIFES and Sync RF+ energies on submental volume. Additionally, it evaluates the effect of HIFES and Sync RF+ on submental fat volume, and HIFES and Sync RF on cheek fat volume.

## METHODS

Thirty-seven subjects (n = 37, 36 female patients and 1 male patient) were enrolled in this multicenter, prospective, open-label study. The inclusion criteria were: adults, clearly visible excess fat in the submentum, willingness and ability to abstain from partaking in any facial treatments other than the study procedure during study participation, complying with study instructions, and consenting to have their face and neck photographed. The exclusion criteria were: local bacterial or viral infection in the treated area, isotretinoin- and tretinoin-containing medication use in the past 12 months, skin-related autoimmune diseases, pregnancy/nursing or in vitro fertilization procedure, acute neuralgia and neuropathy, or any surgical procedure in the treatment area within the last 3 months or before complete healing. Subjects who met the inclusion criteria and none of the exclusion criteria were enrolled, and educated about the study procedures. Written consent was provided, by which the patients agreed to the usage and analysis of their data. This study followed an IRB (Advarra, Pro00070110) approved study protocol that conformed to the ethical guidelines of the 1975 Declaration of Helsinki, and was registered at the clinicaltrials.gov site (NCT06274177). The study was conducted between July 2023 and March 2024.

All subjects received concurrent treatments on the submental and cheek area using self-adhesive applicators (EMFACE, BTL Industries Inc., Boston, MA). The submental applicator delivered HIFES and Sync RF+ to the submental area, while the cheek applicators applied HIFES and Sync RF to the cheeks. The treatment protocol consisted of four 20-minute treatments, spaced 5 to 10 days apart. Intensities of HIFES and Sync RF on the cheeks, and HIFES and Sync RF+ on the submentum were set on maximally tolerated levels according to the subject's feedback. Follow-up visits were scheduled at 1 and 3 months after the last treatment. To rule out submental volume changes through weight fluctuations, subjects were instructed to adhere to their prestudy diet and exercise routine. Body weight was measured at baseline, and at the 1- and 3-month follow-up visits.

### Data Acquisition

MRI scans were conducted at baseline and at both follow-up visits. Sagittal and cranial MRI scans of the head and neck in Dixon sequence were obtained, with participants lying supine and instructed to breathe through their nose. The chin, tip of the nose, and glabella were aligned with the long axis of the MRI scanner, and the lateral canthi and superior margins of the ears were aligned with the transverse axis of the scanner. Two-dimensional (2D) and three-dimensional (3D) photographs acquired with a Vectra H2 camera (Canfield Scientific, Inc., Parsippany, NJ) or a LifeViz Mini (QuantifiCare SA, Biot, France) were taken at baseline, after the last treatment, and at both follow-ups.

Subject satisfaction was assessed with a 5-point Likert scale questionnaire, consisting of the following questions: “My under-chin feels toned and lifted after the treatments,” “After the treatments, the lax skin under my chin has improved,” “My jawline looks more defined after the treatments,” “My overall facial appearance has improved,” “I am satisfied with the treatment results.” This questionnaire was given to participants after the last treatment and at both follow-up visits, while therapy comfort was determined with a 5-point Likert scale questionnaire, which includes a 10-grade numerical rating scale for pain and the statement “I found the treatment comfortable.” Monitoring for adverse events and side effects took place throughout the whole study.

### Evaluations

**Figure 1.**
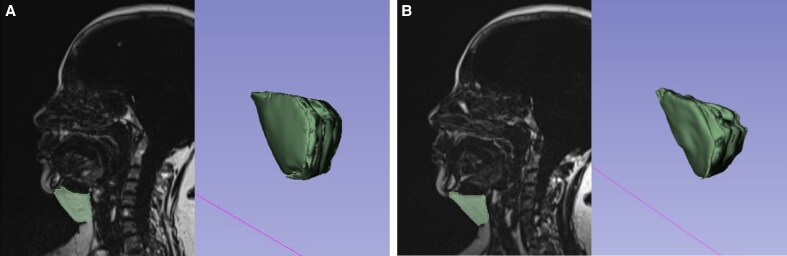
Illustration of submental fat volume analysis from MRI scans. (A) Baseline. (B) One month posttreatment. The left image within both parts displays the submental fat region marked by the radiologist on the MRI scan, while the right image in both parts presents the corresponding 3D model generated from the marked fat. The software then calculates the fat volume based on this 3D reconstruction.

Only eligible data of adequate quality were included in the evaluation. The change in submental fat thickness was assessed by identifying the thickest part of the submental fat layer on the midsagittal plane at the baseline scan and comparing this reference point to corresponding measurements on follow-up scans. To avoid distortion of the results by the position in which the patient lay, and for higher accuracy, changes to the treated area were additionally assessed as changes in the volume. The change in volume and thickness of the submental and cheek area was examined on MRI scans using the semiautomated software 3D Slicer. Calculation of the relative change in the fat layer volume of the cheeks and submental area was performed, as well as the overall submental volume, consisting of the skin, subcutaneous fat tissue, and ABDM. The submental area was limited superiorly by the mylohyoid muscle, anteriorly by the mandible, and inferiorly by the inferior border of the submental fat (Figure 1). The examined cheek fat volume involved the bilateral summation of superficial cheek fat compartments consisting of medial cheek fat, nasolabial fat, middle cheek fat, and lateral cheek fat.

Additionally, 3D image analysis software (Quantificare Inc. or Vectra by Canfield Scientific, Inc.) was used to quantify the submental area's volume from the obtained 3D photographs. The software constructed a 3D model of the subject, and the evaluator marked the overall area of the submental region. This marked area was then used to determine the volume change following the study treatment.

From examination of the 2D photographs, assessment of submental volume severity was performed by 3 independent clinicians based on the 4-grade Clinician-Reported Submental Fat Rating Scale (CR-SMFRS), where 0 means “no localized submental fat evident” and 4 means “extreme submental convexity.” An improvement was considered a decrease in the score.

Descriptive statistics (mean value, standard deviation) and statistical analysis were calculated with GraphPad Prism software (GraphPad Software, Boston, MA). Due to some missing values, a mixed-effects model was applied for the statistical analysis of changes throughout the study. Pairwise comparisons were processed using Tukey's multiple-comparison test. The level of statistical significance was considered to be a = 0.05.

## RESULTS

Thirty-seven subjects (n = 37, 36 females and 1 male; mean [standard deviation] age, 42.2 [12.4] years; BMI, 28.2 [7.2] kg/m^2^; Fitzpatrick skin type, II-V) enrolled in this study and attended the baseline visit. Thirty-three subjects (n = 33, 32 females and 1 male; age, 42.6 [11.9] years; BMI, 28.7 [5.6] kg/m^2^; Fitzpatrick skin type, II-V; see Table 1) completed all study treatments and both follow-up visits. The average follow-up time was 3 months. Four subjects in total were withdrawn in the course of the study: 1 subject (n = 1) wished to discontinue their participation, 1 subject (n = 1) dropped out due to scheduling conflicts, 1 subject (n = 1) dropped out without a given reason, and 1 subject (n = 1) had to discontinue participation due to a claustrophobic experience during MRI scanning at the baseline visit. No side effects, complications or treatment-related adverse events were observed during the study.

**Table 1.** Demographic Information of Patients Who Completed Study Treatments and Both Follow-up Visits

Fitzpatrick skin type (n)				Gender (n)		Age (years)^a^	BMI (kg/m^2^)^a^
II	III	IV	V	Male	Female		
10	13	8	2	1	32	42.6 [11.9]	28.7 [5.6]

^a^Mean [standard deviation].

### Volumetric Evaluation of MRI Scans

**Figure 2.**
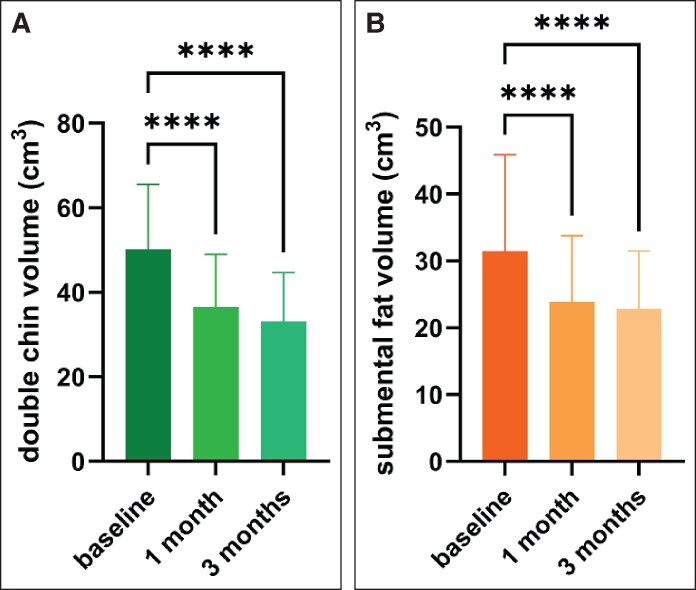
(A) Volume change of the overall submental volume across time as analyzed from MRI scans. (B) Volume change of the submental fat across time as analyzed from MRI scans.

**Figure 3.**
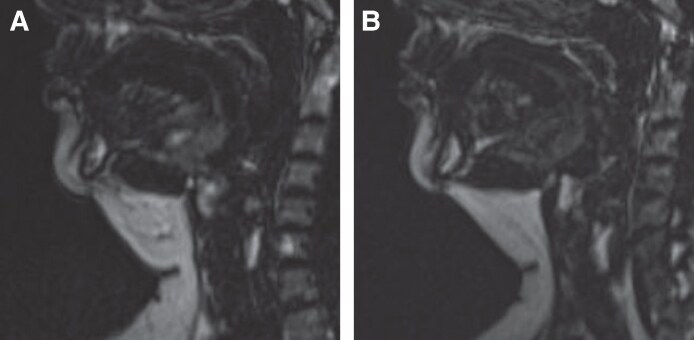
MRI scans of a 55-year-old female patient. This patient underwent combined HIFES and SyncRF+ treatment for submental volume reduction. (A) Baseline. (B) One month posttreatment with a reduction of submental fat layer (white color). The patient reported satisfaction with results, improvement in overall facial appearance, skin laxity, and jawline definition.

Four baseline MRI scans, eight 1-month scans, and seven 3-month MRI scans were unsuitable for evaluation due to motion artifacts or technical errors; therefore, MRI scans of 30 subjects were evaluated for the baseline data, 26 subjects for 1-month data, and 27 subjects for 3-month data. The average submental fat thickness was 15.76 mm at baseline, which reduced by 3.19 mm at 1 month, and by 4.84 mm at 3 months (*P* < .0001). Mean baseline overall submental volume was 50.16 cm^3^, of which the submental fat occupied 31.46 cm^3^. Both the overall submental volume and submental fat decreased following the study treatment (*P* < .0001). The overall volume decreased by 25.12% (mean volume, 36.55 cm^3^, *P* < .0001) at 1 month posttreatment. The submental fat decreased by 20.54% (mean volume, 23.89 cm^3^, *P* < .0001). The changes peaked at 3 months with a reduction of 36.20% (mean volume, 33.09 cm^3^, *P* < .0001) for total volume and 30.37% (mean volume, 22.85 cm^3^, *P* < .0001) for submental fat, as shown in Figures 2 and 3. The change in the cheek fat was not significant, with no notable alteration observed (*P* = .8011), and a difference of 0.07% at the 1-month follow-up and 0.30% at the 3-month follow-up (mean bilateral volumes were 92.25 cm^3^ at baseline, 94.36 cm^3^ at 1 month, and 95.90 cm^3^ at 3 months).

### Volumetric Evaluation of 3D Photographs

Due to 3D imaging artifacts, 4 photographs were excluded from the evaluation of the fourth treatment and 3-month data, and 6 photographs from the 1-month data. For the 3D photograph, 30 subjects were included in the evaluation of the baseline and 3-month data, and 28 subjects for the 1-month data. The evaluation revealed a mean submental volume reduction (*P* < .0001) of 3.48 [3.60] mL after the fourth treatment, 5.39 [5.93] mL at 1 month posttreatment, and 10.25 [5.40] mL at 3 months posttreatment. The differences between the mean values after the fourth treatment and 3-month data, and between the 1-month and 3-month data were statistically significant (*P* < .0001).

### CR-SMFRS Evaluation

**Figure 4.**
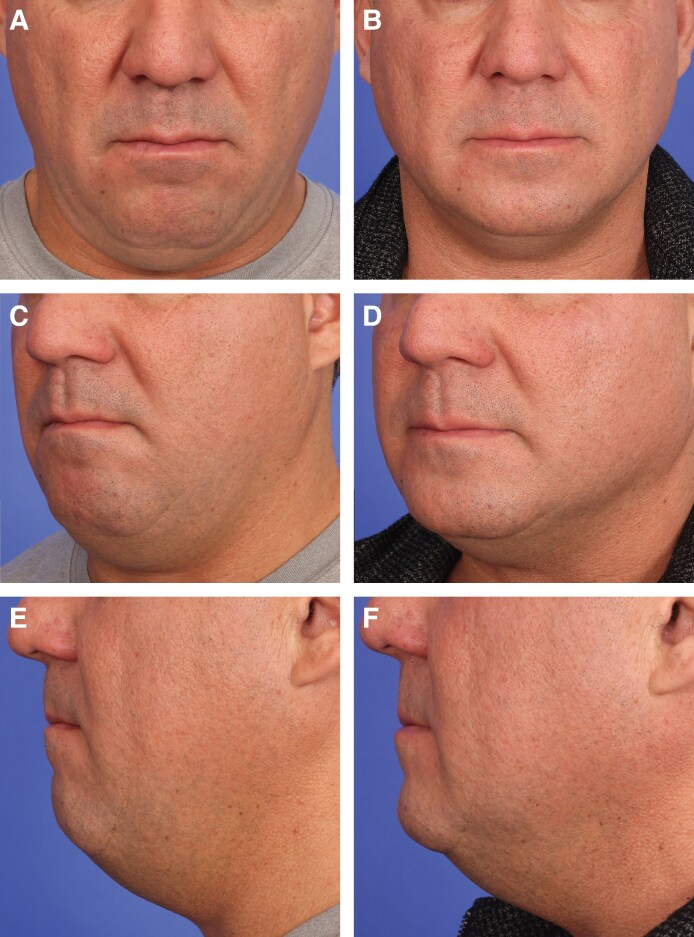
Before-and-after photographs of a 47-year-old male patient with skin type IV following combined HIFES and SyncRF+ treatment for submental volume reduction. (A, C, E) Baseline. (B, D, F) Three months posttreatment with significant double-chin reduction and visible improvement in jawline and chin definition. The change in the BMI was minimal with a reduction of 0.94%. The patient found the treatment comfortable and was satisfied with the results.

**Figure 5.**
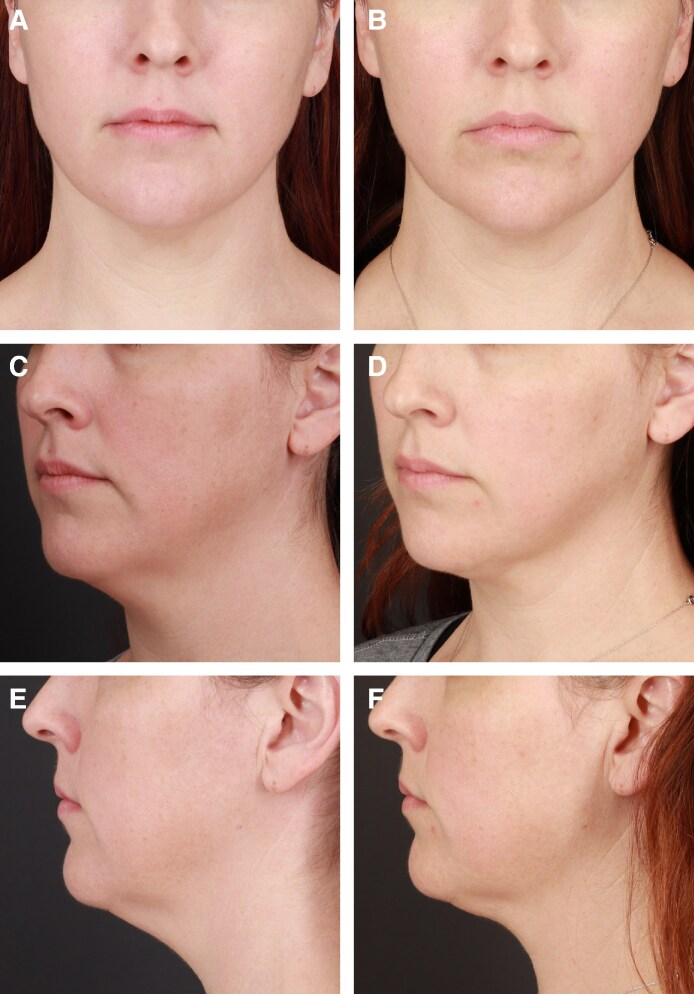
Before-and-after photographs of a 41-year-old female patient with skin type II seeking a double-chin reduction. (A, C, E) Baseline. (B, D, F) Three months posttreatment following combined HIFES and SyncRF+ treatment for submental volume reduction. The patient showed a BMI change of −1.14% and reported an improvement in overall facial appearance.

**Figure 6.**
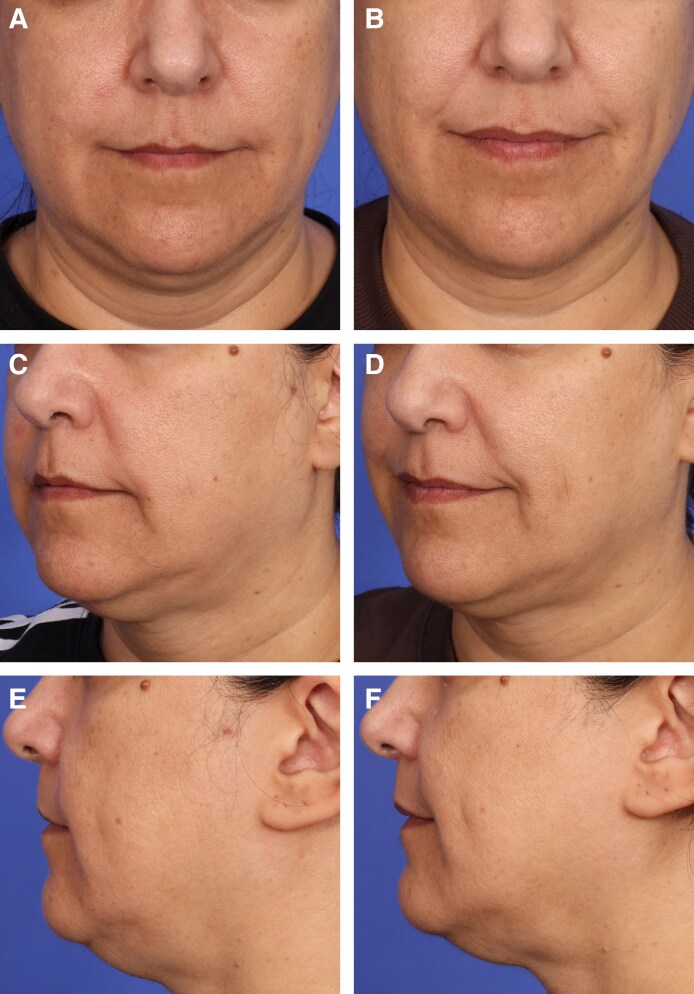
Before-and-after photographs of a 51-year-old female patient with skin type II seeking double-chin reduction. (A, C, E) Baseline. (B, D, F) Three months posttreatment following combined HIFES and SyncRF+ treatment for submental volume reduction. The patient showed a BMI change of −0.37% and reported satisfaction with the results, with her underchin feeling more toned and lifted.

Photographs of 1 subject taken at the 3-month follow-up were excluded from the evaluation because the treated area was covered with clothing during imaging. CR-SMFRS evaluation, therefore, involved 33 subjects for baseline and 1-month data, and 32 subjects for 3-month data. The mean baseline CR-SMFRS grade was 2.33 [0.95] points. With each follow-up, the mean CR-SMFRS grade improved (*P* < .0001), by 0.56 [0.42] points after the fourth treatment (1.82 [0.74] points, *P* < .0001), by 0.85 [0.53] points at 1-month follow-up (1.53 [0.75] points, *P* < .0001), and by 1.03 [0.50] points at 3 months (1.36 [0.79] points, *P* < .0001), as shown in Figures 4-6. Hence, following the treatment, the mean CR-SMFRS grade improved from moderate submental convexity to mild convexity.

### Questionnaires and Weight Measurements

The weight change throughout the study was statistically nonsignificant: −0.37% [4.12%] (*P* = .8957) at 1 month and 0.15% [4.93%] at 3 months (*P* = .9573) compared with baseline.

According to the therapy comfort questionnaire, 84.8% of participants found the treatment comfortable and the average score on the numerical rating scale for pain was 2.8 out of 10. The satisfaction questionnaire revealed that 93.9% of subjects were satisfied with the treatment results, 90.9% reported their underchin feeling more toned and lifted and overall facial appearance improved, and 87.9% found their jawline looking more defined and their skin laxity improved.

## DISCUSSION

The present research aimed to explore the influence of HIFES and Sync RF+ energies on the volume of the submental area. Additionally, it evaluated the effect of HIFES and Sync RF+ on the submental fat volume, as well as HIFES and Sync RF on cheek fat volume. Therapy comfort and subjects’ satisfaction was recorded, as well as safety of the procedures. Submental volume reduction following treatment with HIFES and Sync RF+ was observed on MRI scans and 3D photographs. Although the volume in the submental area treated with HIFES and Sync RF+ decreased, the change in the fat volume of the cheek area was insignificant after the HIFES and Sync RF procedure. Additionally, elevation of the ABDM's position was observed during the MRI evaluation. The treatment was perceived as comfortable and the majority of participants self-reported satisfaction with the study results. No adverse events were reported or observed during the study.

The greater reduction in overall submental volume compared with submental fat volume, as measured by MRI scans, suggests that HIFES and Sync RF+ treatment not only reduces subcutaneous fat tissue but also impacts the skin tissue and the ABDM. The skin tightening following the study procedure, subjectively confirmed by participants, is in alignment with previous research on HIFES and Sync RF technology, where a soft tissue lifting effect of 23% and a 36.6% improvement in wrinkles were reported.^11,12^ The observed change of the ABDM position in this study suggests muscle tone improvement. Previous studies have recognized the ABDM as a contributor to the submental volume,^4,22,23^ although the mechanism is not agreed upon. McCleary et al explored the volume change of this muscle with increasing age, describing an association between age and significantly decreased muscle volume,^4^ in contrast to studies implying bulkiness of the ABDM.^22,23^ The authors suggest muscle laxity and subsequent prolapse as the mechanism of increased submental volume. Further studies are recommended to understand the extent of the ABDM elevation, as well as its volume change, which is beyond the scope of this study.

MRI and 3D image analysis yielded different volume reduction values. The different methodology can explain the variance. MRI volume evaluation measured an anatomically larger area, whereas 3D image analysis examined solely the area covered by the submentum applicator during treatments. Consequently, it was predictable that MRI analysis would indicate a greater volume. While MRI evaluation provides a more accurate and comprehensive understanding of the HIFES and Sync RF+ treatment, the volume reduction from 3D photographs represents the clinical effect, observable by the patient and their acquaintances.

Fat volume in the cheek area remained unaffected following treatment with HIFES and Sync RF, as indicated by a nonsignificant change of 0.07% to 0.30% in the analysis. In contrast, HIFES with Sync RF+ effectively reduced subcutaneous fat tissue in the submental area (by 30.37% at 3 months). This implies the safety of the cheek applicator for facial treatments, considering that fat loss in the face, which is often the result of aging, is undesirable for facial rejuvenation.^3^ The difference in fat volume reduction may be attributed to the varying RF power levels of the 2 applicators. The cheek applicator (HIFES and Sync RF) operates at a lower power than the submental applicator (HIFES and Sync RF+), preventing it from reaching the temperatures required to induce fat apoptosis.^18^ However, further research comparing these 2 modalities on the same treatment area is recommended to confirm this.

Although the study has successfully demonstrated the efficacy and safety of the study procedure, it had certain limitations, including the lack of a control group and limited generalizability of results. Future research would benefit from including a control group, more male subjects, and a wider BMI range. The study, however, included the target population for this procedure. The main strength of this study lies in the objective volumetric measurements based on MRI scans and 3D photographs, as well as the aesthetic improvement assessment based on 2D photographs.

## CONCLUSIONS

Taken together, the study findings suggest the novel approach described is a useful option for submental volume reduction, indicating that HIFES and Sync RF+ technology is capable of aesthetic enhancement as well as skin and muscle improvement, offering a noninvasive approach for submental contouring.
